# Th Cell Phenotypes and Their Correlations with Disease Activity in Patients with Rheumatoid Arthritis

**DOI:** 10.3390/jcm14124220

**Published:** 2025-06-13

**Authors:** Meilang Xue, Haiyan Lin, Tom Lynch, Vibhasha Chand, Premarani Sinnathurai, Ranjeny Thomas, Helen Keen, Catherine Hill, Susan Lester, Mihir Wechalekar, Marissa Lassere, Lyn March

**Affiliations:** 1 Sutton Arthritis Research Laboratory, Sydney Musculoskeletal Health, Kolling Institute, Faculty of Medicine and Health, The University of Sydney, Sydney, NSW 2065, Australia; 2 The Australian Arthritis and Autoimmune Biobank Collaborative (A3BC), Sydney Musculoskeletal Health, Kolling Institute, Faculty of Medicine and Health, The University of Sydney, Sydney, NSW 2065, Australia; 3 Frazer Institute, Translational Research Institute, The University of Queensland, Brisbane, QLD 4102, Australia; 4 Medical School, The University of Western Australia, Perth, WA 6000, Australia; 5 Department of Rheumatology, Fiona Stanley Hospital, Perth, WA 6150, Australia; 6 Adelaide Medical School, The University of Adelaide, Adelaide, SA 5000, Australia; 7 Rheumatology Research Group, Paediatrics and Paediatric Rheumatology, Basil Hetzel Institute, The Queen Elizabeth Hospital, Adelaide, SA 5011, Australia; 8 Rheumatology Synovial Tissue Translational Research Group, Flinders University, Adelaide, SA 5042, Australia; 9 Rheumatology Unit, Flinders Medical Centre, Adelaide, SA 5042, Australia; 10 St George Hospital, School of Population Health, The University of New South Wales, Sydney, NSW 2033, Australia; marissa.lassere@health.nsw.gov.au; 11 Department of Rheumatology, Royal North Shore Hospital, Sydney, NSW 2065, Australia

**Keywords:** rheumatoid arthritis, Th cells, C-reactive protein, disease activity, erythrocyte sedimentation rate, anti-cyclic citrullinated peptide, rheumatoid factor

## Abstract

**Objectives**: This study compared the frequencies of circulating CD4+ T helper (Th) cell subsets in rheumatoid arthritis (RA) patients and healthy controls (HCs) and investigated their relationship with RA disease activity. **Methods**: Peripheral blood samples and demographic/clinical data were collected from 75 RA patients and 28 HCs from the A3BC Biobank. Flow cytometry was utilized to identify cell subsets. Data were analyzed using FlowJo and GraphPad Prism. **Results**: RA patients displayed altered Th cell subset frequencies compared with HCs, including a higher overall proportion of Th cells (*p* = 0.02) but lower proportions of memory Th (*p* = 0.03), Th1 (*p* < 0.0001), and Th17.1 (*p* = 0.004) cells. In DMARD-naïve RA patients (*n* = 16), lower proportions of Th1 (*p* = 0.0005), Th9 (*p* = 0.04), and Th17.1 (*p* = 0.003) cells, alongside a higher Th17 cell proportion (*p* = 0.017), were observed compared with those in HCs. Further analysis of matched treatment-naïve, new-onset RA patients and HCs confirmed these findings. Within the RA cohort, lower proportions of Th1 (*p* = 0.002) and Th17.1 (*p* = 0.025) cells and a higher proportion of Th2 cells (*p* = 0.015) were correlated with increased disease activity. Inverse correlations were also found between the proportions of Th1 (*p* = 0.002), Th9 (*p* = 0.024), and Th17.1 (*p* = 0.00017) cells and CRP levels in RA patients. **Conclusions**: This study demonstrates an imbalance in circulating Th cell subset frequencies in RA patients compared with those in HCs, with notably lower Th1 and Th17.1 cell proportions in RA patients. Decreased frequencies of these cell subsets were linked to increased disease activity, indicating that restoring the balance of Th cell subsets could be a potential therapeutic strategy for RA.

## 1. Introduction

CD4+ T helper (Th) cells play a crucial role in directing immune responses against infections and are also involved in the development of inflammatory and autoimmune diseases [1]. These cells can differentiate into distinct subsets, each with specialized functions and signature cytokines. For instance, Th1 cells primarily combat viruses and intracellular pathogens, while Th2 cells target extracellular parasites, and Th17 cells play a vital role in protecting us from fungal infections and extracellular bacteria.

The delicate balance of the immune system relies on the interaction among these cell subsets. Th1, Th9, Th17, and Th22 cells secret pro-inflammatory cytokines such as TNFα, IFNγ, IL-6, IL-9, IL-17, and IL-22 [2,3], fueling the immune response. In contrast, Th2 and Treg cells produce anti-inflammatory cytokines such as IL-4, IL-13, TGFβ, and IL-35 [4,5], which are essential for maintaining immune homeostasis and preventing the immune system from attacking the body’s own tissues (self-tolerance). Beyond these, T follicular helper (Tfh) cells, comprising Tfh1, Tfh2, and Tfh17 subsets [6], are essential for the activation and maturation of B cells in germinal centers [7]. Conversely, Treg cells, including memory effector Treg cells and T follicular regulatory (Tfr) cells [7] can finely tune germinal center reactions and suppress excessive antibody production [8]. The balanced proportion and function of these Th cell subsets are vital for robust immune regulation and overall health. The disruption of this balance is associated with various inflammatory conditions, including rheumatoid arthritis (RA).

RA is a chronic autoimmune disease characterized by persistent inflammation, leading to the destructive formation of synovial pannuses and progressive joint destruction. Although the precise etiology of RA remains elusive, it is widely accepted that T lymphocytes, particularly Th cells are highly relevant in RA [4,5], in which their normal balance is significantly disrupted, favoring pathogenic phenotypes [9]. Specifically, Th1 and Th17 cells, along with their associated inflammatory cytokines, are often found to be elevated in RA patients [2,10] and show clear correlations with increased inflammation and disease activity [11,12,13]. Conversely, Th2 cells have demonstrated protective effects against arthritis in some models [14,15]. Interestingly, their anti-inflammatory cytokines, IL-4 and IL-13, tend to be upregulated in the early stages of RA but significantly reduced as the disease progresses [16]. Similarly, circulating Treg cells, critical for immune suppression, are often lower in early RA [17] and inversely correlate with disease activity [18]. This profound shift in the balance of Th cell subsets is thought to be a key contributor to both the onset and progression of RA [19,20]. Given this, strategies aimed at restoring the natural, healthy balance of Th cell subsets represent a promising therapeutic approach for RA. In this study, we investigated the profiles of circulating Th cell subsets in patients with RA and thoroughly examined how these specific subsets correlate with various measures of disease activity.

## 2. Materials and Methods

Study participants: This study included 75 established RA patients and 28 healthy controls (HCs). The RA patients met the 2010 American College of Rheumatology/EULAR classification criteria [21], while the HCs had no known history of autoimmune diseases. Peripheral blood mononuclear cells (PBMCs) were collected at a single timepoint from the participants through The Australian Arthritis and Autoimmune Biobank Collaborative (A3BC). Demographic and clinical data, including age, sex, disease duration, smoking status, C-reactive protein (CRP), erythrocyte sedimentation rate (ESR), anti-cyclic citrullinated peptide (anti-CCP) status, rheumatoid factor (RF) status, and disease activity measures (28/66 Swollen Joint Count [SJC], 28/68 tender joint count [TJC], DAS28-CRP, and DAS28-ESR), were also obtained. The relevant characteristics of the RA patient cohort are detailed in Supplementary Tables S1 and S2. At the time of blood sampling, the RA patient group comprised 16 disease-modifying antirheumatic drug (DMARD)-naïve individuals (including 2 with disease durations of 0.5 and 2 years and the remainder with new-onset RA), 12 patients receiving only conventional synthetic (cs)DMARD therapy, and 8 patients receiving both csDMARD and a biologic or targeted synthetic (b/ts)DMARD only. Furthermore, 1 patient received only corticosteroid therapy, 6 patients received only non-steroidal anti-inflammatory drugs, 11 patients received three treatments, 5 patients received all treatments, and 6 patients received no treatment (Supplementary Table S2).

The use of human specimens and patient clinical data was approved by the Northern Sydney Local Health District Human Research Ethics Committee (protocol code 2023/ETH02455 and date of approval 1 November 2023) and the A3BC (A3BC Access ID: ResID1-PID2). All the patients provided their written informed consent.

Flow cytometry: PBMCs were analyzed using flow cytometry (LSR Fortessa cytometer, BD Biosciences, North Ryde, NSW, Australia). Th cells and their subsets were identified using the following antibody panel (all from BD Biosciences): CD194-BV421, CD185-BV480, CD45RA-BV605, CD127-BV711, CD196-BV786, CD25-BB515, CD294 (CRTH2)-PE-CF594; CD183-PE-Cy7, CCR10-APC, CD3-Alexa Fluor 700, CD4-APC-H7. This panel allowed for the identification of Th1, Th2, Th9, Th17, Th17.1 (defined as CCR6+CXCR3+CCR4− within the non-Treg cell population), Th22, Treg cells (memory/effector Treg and Tfr), and Tfh cell subsets (Tfh1, Tfh2, and Tfh17) as previously described [22]. Live and dead cells were distinguished using 7-AAD staining. Flow cytometry data were analyzed using FlowJo v10 software (BD Biosciences, Ashland, OR, USA ).

Quality control steps: Sample quality was assessed using the FlowAI plugin in FlowJo, and only high-quality events were included in the analysis. To ensure robust subset identification, only samples containing at least 50,000 single, live T cells were analyzed, guaranteeing a minimum of 100 cell events per subset. Additionally, a control sample run under identical conditions was included in each experimental batch.

Automated clustering and visualization: The automated clustering of cell populations was performed using the FlowSOM algorithm and visualized with t-distributed stochastic neighbor embedding (tSNE) via FlowJo plugins. Marker enrichment modeling (MEM) was employed to automatically generate quantitative labels for the identified cell clusters, enabling quantitative comparisons.

Statistical analysis: Statistical analyses were conducted using GraphPad Prism 10 (GraphPad Software, Boston, MA, USA). Data are presented as means with a 95% confidence interval (CIs). Non-parametric Mann–Whitney U tests were used for comparisons between the HC and RA groups, as well as within the RA group. Correlations between variables were assessed using the non-parametric Spearman’s rank correlation coefficient test and linear regression test. A two-sided *p* < 0.05 was considered statistically significant.

## 3. Results

### 3.1. Patients and Data Cohort

This study included 75 established RA patients (mean age 54.9 ± 14.8 years; 53 females, 22 males) and 28 HCs (mean age 45.6 ± 15.2 years; 22 females, 6 males). The demographic, pathological, and clinical characteristics of these RA patients are detailed in Supplementary Tables S1 and S2. The RA patients were significantly older than the HCs (*p* = 0.006).

Within the RA cohort, no significant differences were observed between male and female or between seronegative and seropositive patients, regarding age, disease activity measures (SJC, TJC, DAS28-CRP, DAS28-ESR), levels of CRP and ESR, or the proportions of RF or anti-CCP positivity between male and female patients. However, male RA patients had a significantly shorter disease duration (2.0 ± 5.7 years vs. 10.5 ± 11.4 years, *p*= 0.001); lower proportions of Th cells (0.98 ± 0.51 vs. 1.36 ± 0.62, *p* = 0.01), Th17.1 cells (0.43 ± 0.54 vs. 1.17 ± 1.22, *p* = 0.009), and Tfh1 (0.51 ± 0.24 vs. 0.70 ± 0.43, *p* = 0.048); and a higher proportion of Th2 cells (1.79 ± 0.88 vs. 1.15 ± 0.59, *p* = 0.0005) than female RA patients.

### 3.2. Differential Frequencies of Circulating Th Cells and Subsets in RA Patients and HCs

**Figure 1:**
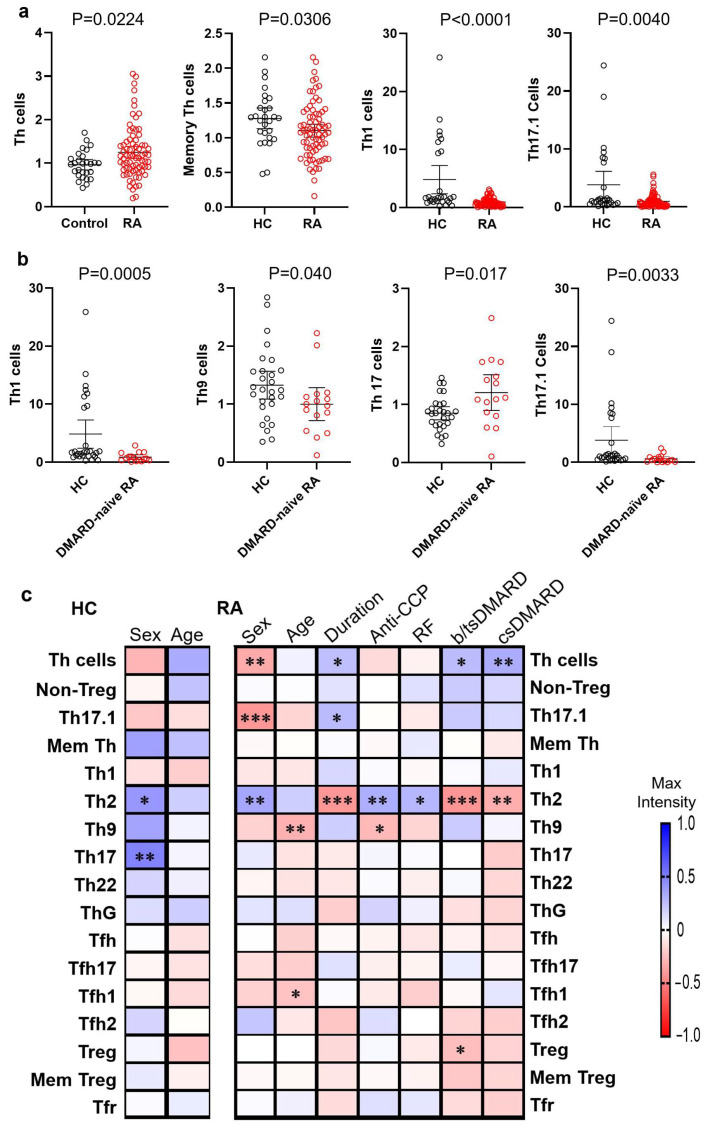
Th cells and their subsets in patients with rheumatoid arthritis (RA) and healthy controls (HCs). Peripheral blood mononuclear cells from RA patients (*n* = 75) and HCs (*n* = 28) were analyzed by flow cytometry to determine the levels of Th cells and their subsets. (**a**) Relative levels of total Th cells, memory Th cells, Th1 cells, and Th17.1 cells in HCs and RA patients. (**b**) Relative levels of Th1, Th9, Th17, and Th17.1 cells in HCs (*n* = 28) and DMARD-naïve RA patients (*n* = 16). Data presented are means with 95% confidence intervals (CIs). Statistical significance was assessed using non-parametric Mann–Whitney U tests. (**c**) Spearman’s rank correlation coefficients showing significant correlations between Th cell subsets and demographical/clinical parameters (sex, age, disease duration, serological status, and DMARD use) in RA patients and HCs. * *p* < 0.05, ** *p* < 0.001, *** *p* < 0.0001. Anti-CCP: anti-cyclic citrullinated peptide antibodies; RF: rheumatoid factor; csDMARD: conventional synthetic disease-modifying antirheumatic drug; b/tsDMARD: biologic or targeted synthetic DMARD; Mem: memory.

Flow cytometry was used to determine the frequencies of circulating Th cell subsets, as detailed in the gating strategy in Supplementary Figure S1. Compared with the HCs (*n* = 28), the RA patients (*n* = 75) exhibited a significantly higher overall frequency of total Th cells (*p* = 0.02) but lower frequencies of memory Th cells (*p* = 0.03), Th1 cells (*p* < 0.0001), and Th17.1 cells (*p* = 0.004) (Figure 1a).

Further analysis comparing Th cell subset frequencies between DMARD-naïve RA patients (*n* = 16, mean age 54.0 ± 16.3 years, 7 males, mean disease duration 12 days) and HCs revealed that the DMARD-naïve RA group had significantly lower frequencies of Th1 (*p* = 0.0005), Th9 (*p* = 0.04), and Th17.1 (*p* = 0.003) cells but higher frequencies of Th17 cells (*p* = 0.017) than the HCs (Figure 1b).

Non-parametric correlation analysis showed that sex significantly influenced the frequencies of Th2 (*p* = 0.033) and Th17 (*p* = 0.009) cells (Figure 1c), with males having more Th2 and Th17 cells in the HCs. In the RA patients, the frequencies of total Th cells and their subsets showed significant associations with sex, age, disease duration, anti-CCP and RF status, and DMARD use (Figure 1c).

### 3.3. Automated Clustering Reveals Distinct Cell Subset Profiles in Early RA

**Figure 2:**
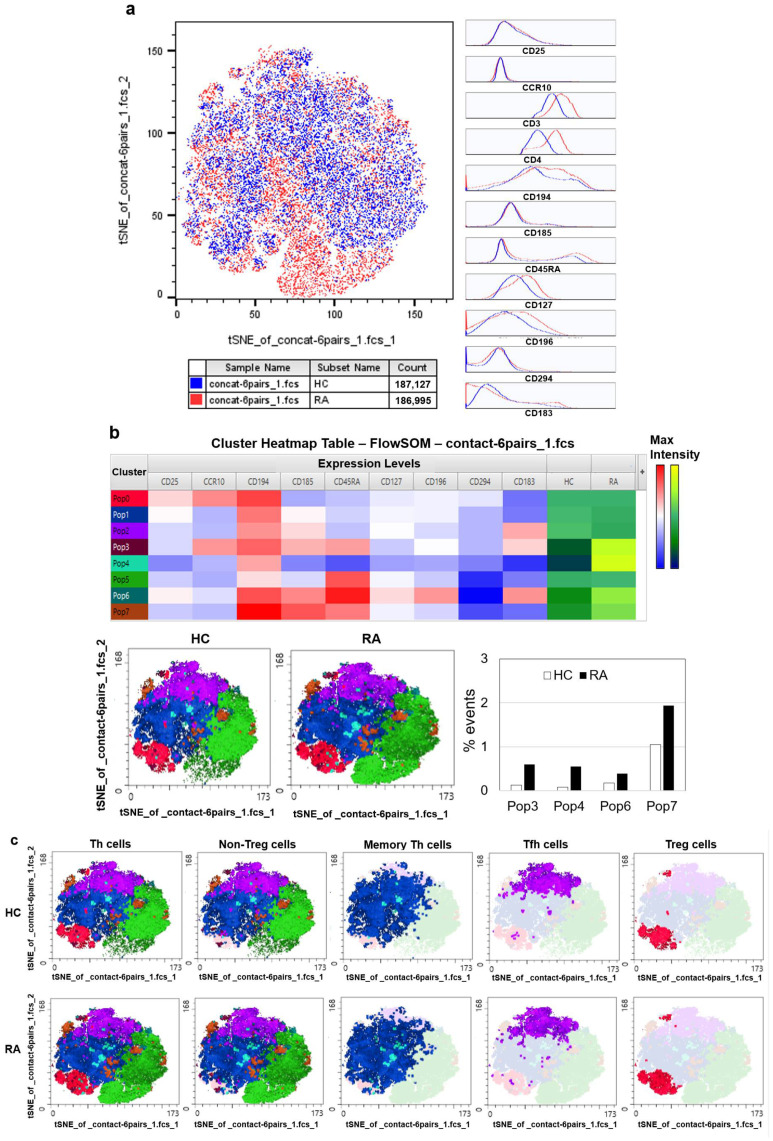
tSNE visualization and automated clustering of Th cell subsets in patients with DMARD-naïve, new-onset rheumatoid arthritis (RA) and healthy controls (HCs). Peripheral blood mononuclear cells from 6 age- and sex-matched pairs of DMARD-naïve, new-onset RA patients and HCs were analyzed by flow cytometry using a T cell antibody panel. (**a**) tSNE plots displaying the expression of CD3, CD4, CD25, CD127, CD183, CD185, CD196, CD294, and CD45RA, with corresponding heatmaps and histograms. Marker expression levels are color-coded on the tSNE plots. (**b**) Cluster analysis identified 4 clusters enriched in RA patients compared with HCs. (**c**) tSNE visualization of Treg cells and non-Treg cells (memory Th and Tfh cells) identified by automated clustering of Th cells. tSNE: t-distributed stochastic neighbor embedding. Tfh cells: T follicular helper cells.

To minimize the influence of confounding factors, tSNE and automated clustering of the Th cell markers were performed on Th cells and memory Th cells from age- and sex-matched DMARD-naïve patients with new-onset RA patients and HCs (*n* = 6 pairs). The tSNE heatmaps and overlaid histograms illustrating Th cell marker expression in these two groups are presented in Figure 2a. Notably, the RA Th cells showed higher expression of CD3, CD4, CD127, CD194, and CD196 compared with the HC Th cells (Figure 2a).

Automated clustering analysis identified cell clusters 3, 4, 6, and 7 as being more abundant in RA patients than in HCs, with population 4 being particularly higher. These populations typically displayed higher or moderate expressions of CD194, CD185, and/or CD45RA (Figure 2b). This analysis also facilitated the clear identification of non-Treg, memory Th, Tfh, and Treg cell subsets (Figure 2c).

**Figure 3:**
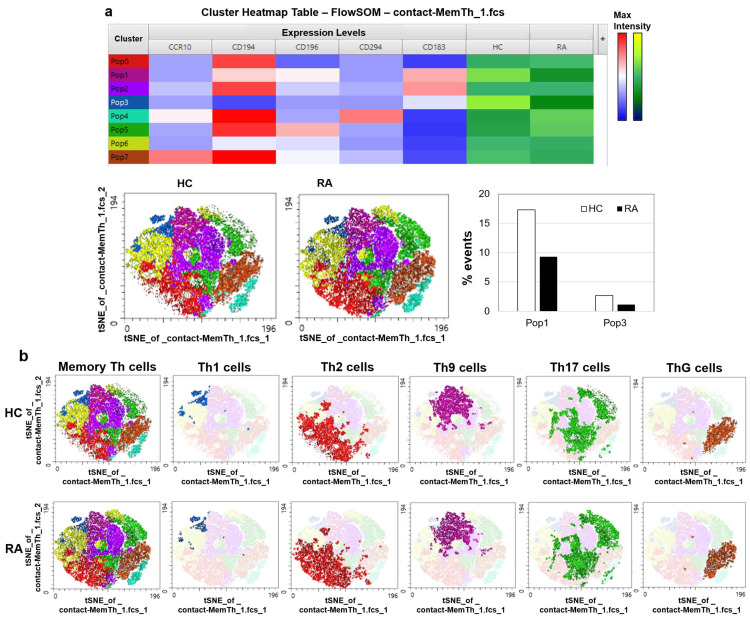
tSNE visualization and automated clustering of memory Th cell subsets in patients with DMARD-naïve, new-onset rheumatoid arthritis (RA) and healthy controls (HCs). Peripheral blood mononuclear cells from 6 age- and sex-matched pairs of DMARD-naïve, new-onset RA patients and HCs were analyzed by flow cytometry using a T cell antibody panel. (**a**) Cluster analysis of memory Th cells identified 2 clusters that were significantly less abundant in RA patients than in HCs. (**b**) tSNE visualization of Th1, Th2, Th9, Th17, and ThG cells identified by automated clustering of memory Th cells. Tfh cells: T follicular helper cells; tSNE: t-distributed stochastic neighbor embedding.

Further automated clustering analysis of memory Th cells, based on the expression of CCR10, CD183, CD194, CD196, and CD294, revealed two specific cell clusters with reduced frequencies in RA patients compared with HCs (Figure 3a). tSNE mapping of memory Th cell subsets, which identified canonical T cell populations (Th1, Th2, Th9, Th17, and ThG), confirmed the lower frequencies of Th1 and Th9 cells (clusters 1 and 3) in RA patients than those in HCs (Figure 3b).

### 3.4. The Correlations of Circulating Th Cells and Their Subsets with RA Disease Activity

**Figure 4:**
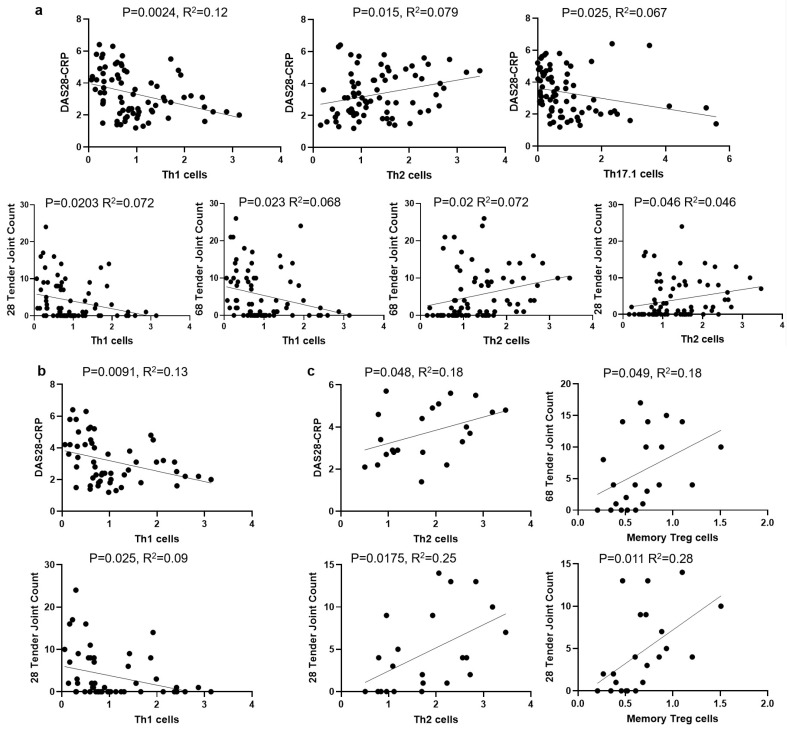
The correlations between Th cell subsets and disease activity in rheumatoid arthritis (RA). Linear regression analysis was used to assess correlations between Th cell subset frequencies and disease activity measures in RA patients. (**a**) The correlations of Th cell frequencies with DAS28-CRP and tender joint count (TJC) in all RA patients (*n* = 75). (**b**) The correlations of Th1 cell frequency with DAS28-CRP and TJC in female RA patients (*n* = 53). (**c**) The correlations of Th2 and memory Th cell frequencies with disease activity in male RA patients (*n* = 22). CRP: C-reactive protein.

Linear regression analysis of the entire RA cohort (*n* = 75) revealed significant correlations between the frequencies of circulating Th cell subsets and disease activity, as measured by DAS28-CRP, TJC, and SJC. Specifically, lower frequencies of Th1 (*p* = 0.002) and Th17.1 (*p* = 0.025) cells and a higher frequency of Th2 cells (*p* = 0.015) were correlated with higher DAS28-CRP scores (Figure 4a). Consistent with these findings, Th1 cell frequency showed a negative correlation with TJC, and Th2 cell frequency showed a positive correlation with TJC (Figure 4a). No significant correlations were observed between Th1 or Th2 cell subset frequencies and SJC.

Subgroup analysis in female RA patients (*n* = 53) showed that a higher Th1 cell frequency was associated with lower DAS28-CRP (*p* = 0.009) and TJC (*p* = 0.025) (Figure 4b). In contrast, in male RA patients, only a higher Th2 cell frequency showed positive correlations with both DAS28-CRP score (*p* = 0.048) and TJC (*p* = 0.018). Notably, memory Treg cell frequency exhibited positive correlations with TJC in male patients (*p* = 0.028, *p* = 0.049) (Figure 4c).

**Figure 5:**
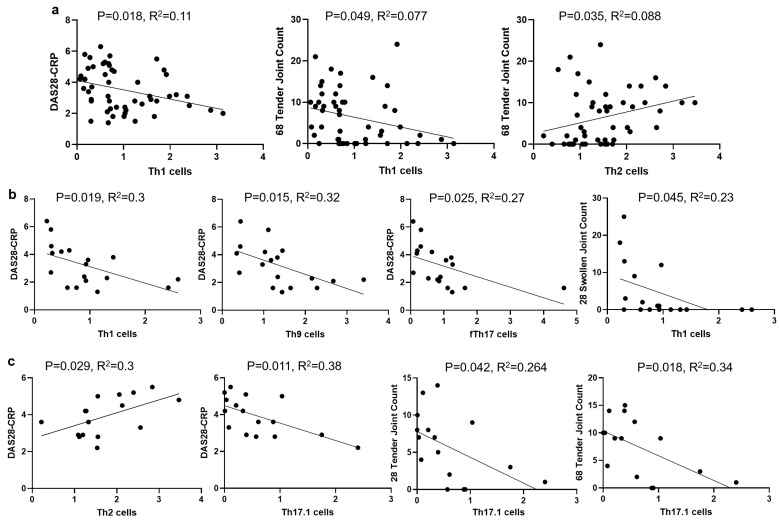
The correlations of Th cell subsets with disease activity in seropositive and seronegative patients and DMARD-naïve patients with rheumatoid arthritis (RA). The correlations of Th cell subsets with disease activity in RA patients were analyzed using a linear regression test. (**a**) The correlations of Th1 and Th2 cell frequencies with disease activity in seropositive patients (*n* = 52). (**b**) The correlations of Th1, Th9, and fTh17 cell frequencies with disease activity in seronegative patients (*n* = 18). (**c**) The correlations of Th2 and Th17.1 cell frequencies with disease activity in DMARD-naïve RA patients (*n* = 16). CRP: C-reactive protein; DMARD: disease-modifying antirheumatic drug.

Stratification by serological status showed that, in seropositive RA patients (*n* = 52), a lower Th1 cell frequency was correlated with increased DAS28-CRP (*p* = 0.018) and 28TJC (*p* = 0.049). Conversely, a higher Th2 cell frequency was associated with increased 68TJC (*p* = 0.035) (Figure 5a). In seronegative RA patients, lower frequencies of Th1, Th9, and fTh17 cells were correlated with increased DAS28-CRP (*p* = 0.019, *p* = 0.015, and *p* = 0.025, respectively) (Figure 5b). Additionally, a lower Th1 cell frequency was associated with increased SJC (*p* = 0.045).

Finally, in DMARDs-naïve patients (*n* = 16), a higher Th2 cell frequency was correlated with increased DAS28-CRP (*p* = 0.03), while a higher Th17.1 cell frequency was correlated with lower DAS28-CRP (*p* = 0.011) and TJC (*p* = 0.042, *p* = 0.018) (Figure 5c).

### 3.5. The Correlations of Th Cell Subsets with CRP and ESR in RA Patients

**Figure 6:**
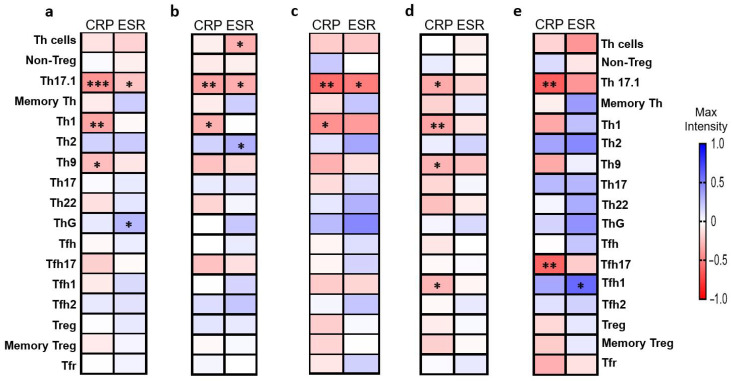
The correlations between Th cell subsets and C-reactive protein (CRP) and erythrocyte sedimentation rate (ESR) in rheumatoid arthritis (RA). Nonparametric correlation matrix analysis was used to assess associations between Th cell subset frequencies and the levels of CRP and ESR in RA patients. (**a**) Overall RA patients (*n* = 75). (**b**) Female RA patients (*n* = 53). (**c**) Male RA patients (*n* = 22). (**d**) Seropositive RA patients (*n* = 52). (**e**) Seronegative RA patients (*n* = 18). * *p* < 0.05, ** *p* < 0.001, *** *p* < 0.0001.

The CRP and ESR levels in RA patients are presented in Supplementary Table S1. Overall, the CRP levels showed significant negative correlations with the frequencies of Th1 (*p* = 0.002), Th9 (*p* = 0.024), and Th17.1 (*p* = 0.00017) cells. ESR was negatively correlated with Th17.1 cell frequency (*p* = 0.044) and positively correlated with ThG cell frequency (*p* = 0.019) (Figure 6a).

In female patients, ESR showed negative correlations with the frequencies of total Th cells (*p* = 0.027) and Th17.1 cells (*p* = 0.019) and a positive correlation with Th2 cell frequency (*p* = 0.029), while CRP was negatively correlated with Th1 (*p* = 0.026) and Th17.1 (*p* = 0.009) cell frequencies (Figure 6b). In male patients, both CRP and/or ESR exhibited negative correlations with the frequencies of Th17.1 (*p* = 0.005 and *p* = 0.015, respectively) and Th1 cells (*p* = 0.04) (Figure 6c).

In seropositive patients, CRP was negatively correlated with the frequencies of Th1 (*p* = 0.01), Th9 (*p* = 0.034), Th17.1 (*p* = 0.017), and Tfh1 (*p* = 0.041) cells (Figure 6d). ESR showed no significant correlations with Th cell subsets in this group. In seronegative patients, CRP was negatively correlated with the frequencies of Th17.1 (*p* = 0.005) and fTh17 (*p* = 0.008) cells, while ESR showed a positive correlation with Tfh1 cell frequency (*p* = 0.011) (Figure 6e). No significant correlations between Th cell subsets and CRP or ESR were observed in the DMARD-naïve patient subgroup.

## 4. Discussion

This study shows significant changes in Th cell subsets in established RA patients, with higher total Th cell frequency but lower Th1 and Th17.1 cell frequencies compared with HCs. Similar trends were observed in new-onset RA patients. Within the RA cohort, higher Th1 and Th17.1 frequencies were correlated with lower disease activity scores (DAS28-CRP, TJC), while higher Th2 frequencies were linked to higher disease activity. Reduced Th1 and Th17.1 frequencies were also associated with higher CRP levels, though ESR correlations varied by subgroup.

These findings align with the established functions of Th cells in RA pathogenesis [2,23], where both numerical and functional abnormalities are observed in circulation and at inflamed joints [2,17,24,25], including the increase in overall Th cell frequency as shown in this study. The finding of reduced frequencies of circulating Th1, Th9, and Th17.1 cells in RA patients compared with HCs challenges the traditional perception of their roles in RA [26], which is often characterized as a Th1-driven disease [3]. In contrast, Th2 cells are often associated with anti-arthritic properties [2]. However, recent studies have also reported decreased Th1 but increased Th2 cells in RA [27,28], with evidence suggesting a potential pathogenic role for Th2 cells in early, untreated RA [28,29]. The observed negative association of Th1 cell and positive association of Th2 cell frequencies with disease activity in our cohort further supports their respective roles in RA. The lower circulating Th1 frequency might also reflect their recruitment to the inflamed synovium, where they likely contribute to the inflammatory cascade, as suggested by higher Th1 proportions in synovial fluid [30]. Future analysis of synovial tissue Th subsets could validate this.

Th9 cells play a dual role in inflammatory diseases by inducing naive Th cells to differentiate into Th17 cells and enhancing the suppressive function of Treg cells [31]. While elevated Th9 and IL-9 have been reported in RA patients’ peripheral blood and synovium [32,33], paradoxically, higher IL-9-producing cells have also been associated with RA remission [34]. Furthermore, Th9 cells have been shown to promote the resolution of arthritis in animal models [34] highlighting the importance of Th9 cells and IL-9 in resolving inflammation associated with RA. Similarly, higher baseline Th17.1 cell numbers have been linked to better treatment response in RA [35], suggesting that these cells may play a role in alleviating inflammation. In our established RA cohort, we noted lower levels of Th9 and Th17.1 cells, which likely reflect ongoing, unresolved chronic inflammation. This observation is consistent with a previous study showing a lower proportion of circulating Th17.1 cells in patients with early RA compared with HCs [36]. The contrasting correlations between Th2 cell frequencies (which are positively associated) and Th17.1 cell frequencies (which are negatively associated) with disease activity in DMARD-naïve patients further highlight their potential functional roles in the pathogenesis of RA.

While seropositive RA is typically associated with higher disease activity and poorer outcomes, seronegative RA is associated with greater inflammatory activity [37]. Our study did not find significant differences in disease activity scores and the proportions of Th cell subsets between these groups, consistent with some previous reports [24,38]. However, we did observe distinct correlation patterns between Th cell subsets and disease activity based on serostatus, suggesting differential roles of these subsets in seronegative and seropositive RA. The negative correlation of Th1 cell frequency with disease activity in seropositive patients and the negative correlation of Th1, Th9, and fTh17 cell frequencies in seronegative patients might reflect compensatory mechanisms in the latter group with potentially higher inflammatory burden [37]. Notably, only Th1 cell frequency correlated with SJC across the entire cohort.

The observed consistent negative correlations between Th1 and Th17.1 cell frequencies and CRP levels in the overall cohort of RA patients are significant. This correlation holds across all subgroups, including male, female, seronegative, and seropositive patients. CRP is a marker of systemic inflammation in RA, and serum CRP levels significantly correlate with knee synovial inflammation scores in RA patients [39]. The negative correlations between CRP levels and the frequencies of Th1 and Th17.1 cells suggest that these cells may play a protective role in RA.

This study has limitations due to its relatively small sample size, which may impact the accuracy of the estimates and the ability to detect subtle effects. Furthermore, the variability in the use of non-DMARD medications complicates our assessment of how these drugs may influence Th cell populations. Consequently, our findings should be regarded as preliminary and require validation in a larger, more diverse cohort.

## 5. Conclusions

This study demonstrates a significant shift in Th cell phenotypes in RA patients compared with HCs, characterized by reduced frequencies of Th1 and Th17.1 cells and increased frequencies of Th and Th2 cells. Notably, RA disease activity (DAS28-CRP) showed negative correlations with Th1 and Th17.1 cell frequencies and a positive correlation with Th2 cells. Additionally, inflammatory markers such as CRP and ESR displayed negative associations with Th1 and Th17.1 cell subsets. These findings support an association between Th subset imbalance and RA pathogenesis. While our data suggest that restoring the natural balance of Th cell subsets may be beneficial for RA management, further functional and longitudinal studies are necessary to confirm whether therapeutic modulation of these populations can indeed influence clinical outcomes. 

## Data Availability

The data supporting this article are available from the corresponding author upon reasonable request.

## Supplementary Materials

The following supporting information can be downloaded at: https://www.mdpi.com/article/10.3390/jcm14124220/s1, Figure S1: The gating strategies for flow cytometric detection of Th cells and their subsets; Table S1: Demographic and clinical characteristics of patients with rheumatoid arthritis; Table S2: Smoking and treatment status of patients with rheumatoid arthritis (RA) and healthy controls (HC).

## Abbreviations

The following abbreviations are used in this manuscript:

Anti-CCP	anti-cyclic citrullinated peptide
b/ts	biologic or targeted synthetic
CI	confidence interval
CRP	C-reactive protein
cs	conventional synthetic
DMARD	disease-modifying antirheumatic drug
ESR	erythrocyte sedimentation rate
HCs	healthy controls
MEM	marker enrichment modeling
PBMCs	peripheral blood mononuclear cells
RA	rheumatoid arthritis
RF	rheumatoid factor
SJC	swollen joint count
Tfh cells	T follicular helper cells
Th cells	CD4+ T helper cells
TJC	tender joint count
Tfr	T follicular regulatory
tSNE	t-distributed stochastic neighbor embedding
