# Supplementary material for: Th Cell Phenotypes and Their Correlations with Disease Activity in Patients with Rheumatoid Arthritis
Source: J Clin Med. 2025 Jun 13;14(12):4220. doi: 10.3390/jcm14124220 (PMC12194616; doi:10.3390/jcm14124220)

Table S1 Demographic and clinical characteristics of patients with rheumatoid arthritis

	Age (years)	Duration (years)	66 SJC	68 TJC	28 TJC	28 SJC	DAS28 -CRP	DAS28 -ESR	Anti-CCP	RF	CRP (mg/dL)	ESR (mm/hr)
Overall (n=75)	54.9±14.8	8.0±10.7	4.9±6.3	5.4±6.7	4.0±5.4	4.0±5.5	3.3±1.4	3.5±1.5 (n=73)	54+/15-	51+/23-	11.2±20.1	22.6±23.5 (n=74)
Females (n=53)	55.5±14.5	10.5±11.4	4.4±6.4	5.2±7.0	3.8±5.6	3.7±5.6	3.2±1.4	3.5±1.6 (n=52)	28+/19-	33+/17-	11.1±18.6	25.8±24.5 (n=52)
Males (n=22, 29%)	53.3±15.8	2.0±5.7	5.2±5.9	4.4±6.2	2.4±2.3	3.2±5.5	3.7±1.3	3.7±1.5	18+/4-	16+/6-	14.0±23.6	15.0±19.2
Seropositive (n=52)	55.8±14.8	6.2±9.0	5.1±5.8	6.3±6.4	4.5±5.0	4.1±4.9	3.5±1.4	3.7±1.6 (n=50)	20+/2-	21+/2-	10.7±18.6	23.3±25.3 (n=51)
Seronegative (n=18)	51.9±14.5	10.6±13.7	5.3±8.3	4.4±7.7	3.6±6.7	4.7±7.5	3.0±1.5	3.4±1.4	_	_	15.6±25.8	22.1±19.5

The data was gathered from The Australian Arthritis and Autoimmune Biobank Collaborative (A3BC), at the time blood was drawn. Data in the table are shown as mean ± SD. Anti-CCP: cyclic citrullinated peptide antibodies; CRP: C-reactive protein; ESR: erythrocyte sedimentation rate; RF: rheumatoid factor; SJC: swollen joint count; TJC: tender joint count. +/–: positive/negative.

Table S2 Smoking and treatment status of patients with rheumatoid arthritis (RA) and healthy controls (HC)

	csDMARD	b/tsDMARD	NSAIDs	Corticosteroids	Smoking
Overall RA (n=75)	56+/17–	30+/42–	38+/25–	24+/46–	10+/39–
Female RA (n=53)	42+/9–	26+/25–	26+/17–	17+/33–	3+/27–
Male RA (n=22)	14+/8–	4+ /16–	12+/7–	7+/13–	7+/12–
Total HC (n=28)	28–	28–	26–	24–	4+/22–
Female HC (n=22)	22–	22–	20–	19–	3+/17–
Male HC (n=6)	6–	6–	6–	6–	1+/5–

The data was gathered from The Australian Arthritis and Autoimmune Biobank Collaborative (A3BC), at the time blood was drawn. csDMARD: conventional synthetic disease-modifying antirheumatic drugs. b/tsDMARD: biologic or targeted synthetic DMARD. NSAIDs: non-steroidal anti-inflammatory drugs. +/–: treated or smoking/not treated or non-smoking.

Figure S1. The gating strategies for flow cytometric detection of Th cells and their subsets.

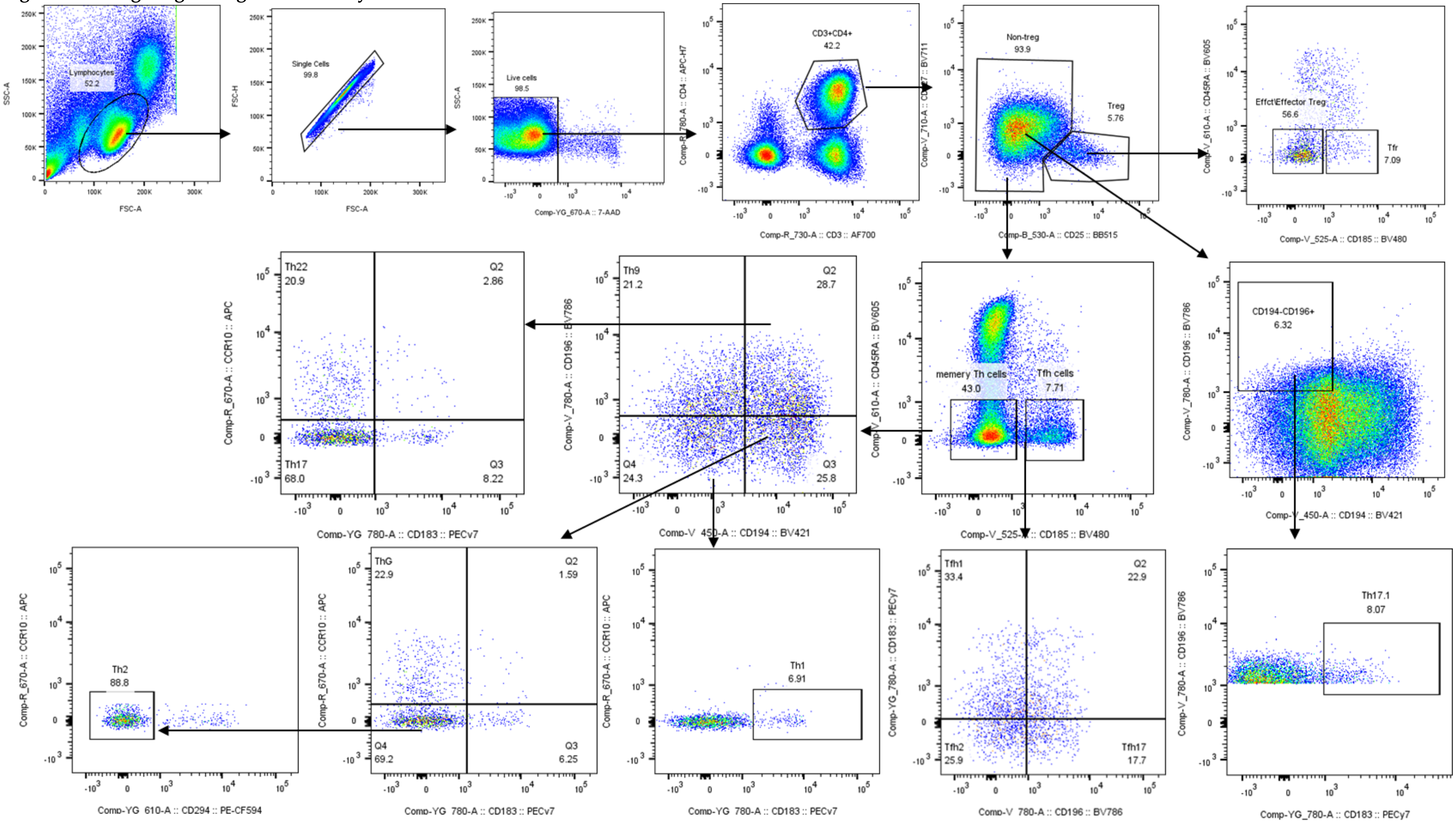

Supplement: Supplementary file 1 [file jcm-14-04220-s001.zip › jcm-3646137-supplementary.pdf]
